# Nonmicrosurgical reconstruction of the auricle after traumatic amputation due to human bite

**DOI:** 10.1186/1746-160X-2-45

**Published:** 2006-12-01

**Authors:** Dionysios E Kyrmizakis, Alexander D Karatzanis, Constantinos A Bourolias, John K Hadjiioannou, George A Velegrakis

**Affiliations:** 1 Department of Otolaryngology, University of Crete, School of Medicine, Heraklion, Crete, Greece

## Abstract

**Background:**

Traumatic auricular amputation due to human bite is not a common event. Nonetheless, it constitutes a difficult challenge for the reconstructive surgeon. Microsurgery can be performed in some cases, but most microsurgical techniques are complex and their use can only be advocated in specialized centers. Replantation of a severed ear without microsurgery can be a safe alternative as long as a proper technique is selected.

**Methods:**

We present two cases, one of a partial and one of a total traumatic auricular amputation, both caused by human bites, that were successfully managed in our Department. The technique of ear reattachment as a composite graft, with partial burial of the amputated part in the retroauricular region, as first described by Baudet, was followed in both cases.

**Results and discussion:**

The prementioned technique is described in detail, along with the postoperative management and outcome of the patients. In addition, a brief review of the international literature regarding ear replantation is performed.

**Conclusion:**

The Baudet technique has been used successfully in two cases of traumatic ear amputation due to human bites. It is a simple technique, without the need for microsurgery, and produces excellent aesthetic results, while preserving all neighboring tissues in case of failure with subsequent need for another operation.

## Background

The traumatic loss of an ear constitutes a great aesthetic deformity and considerably affects the patient's psychology. In addition, the severed ear constitutes a major challenge for the head and neck or plastic surgeon particularly when a human bite is the cause, taking into account the high possibility of severe contamination by the bacteria of oral flora. The difficulty of reconstitution is mainly related to the unique anatomical structure of the auricle, with fine skin covering, a thin and elastic cartilage, and small size vessels responsible for its perfusion [1,2].

Many microsurgical techniques have been reported for reattachment of the auricle, but their significant complexity and numerous limitations do not allow for wide practice [1-3]. On the other hand, simple reattachment of the amputated part as a composite graft is doomed to fail with almost certainty [1,4]. Therefore, numerous techniques that increase the chance of survival of the replanted ear segment have evolved in the past [1,4,5].

In 1972, Baudet et al, reported a case of successful ear replantation using a novel technique. Reattachment was accomplished by excising the posterior skin of the amputated part and making large fenestrations in the cartilage to allow better contact of the anterior skin to the underlying vascular bed. In addition, a postauricular flap was elevated. The anterior skin was then sutured to the amputated stump of the ear and to the postauricular flap. In this way, a larger area of inset and greater surface of contact with the vascular bed was provided for the graft, thus allowing for better composite graft survival [6].

No cases of reconstruction of traumatic auricle amputation have been published so far in ENT literature. In this report, we describe our experience with the use of the prementioned technique in two cases, one of a partial and one of a total traumatic ear amputation due to human bites, followed by a review of the international literature.

## Methods

### Case 1

**Figure 1:**
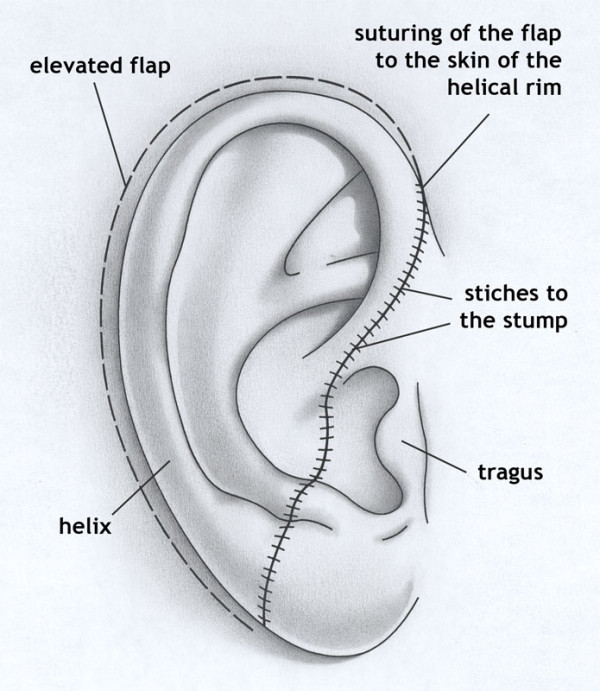
A diagram depicting the basic principles of the Baudet technique.

**Figure 2:**
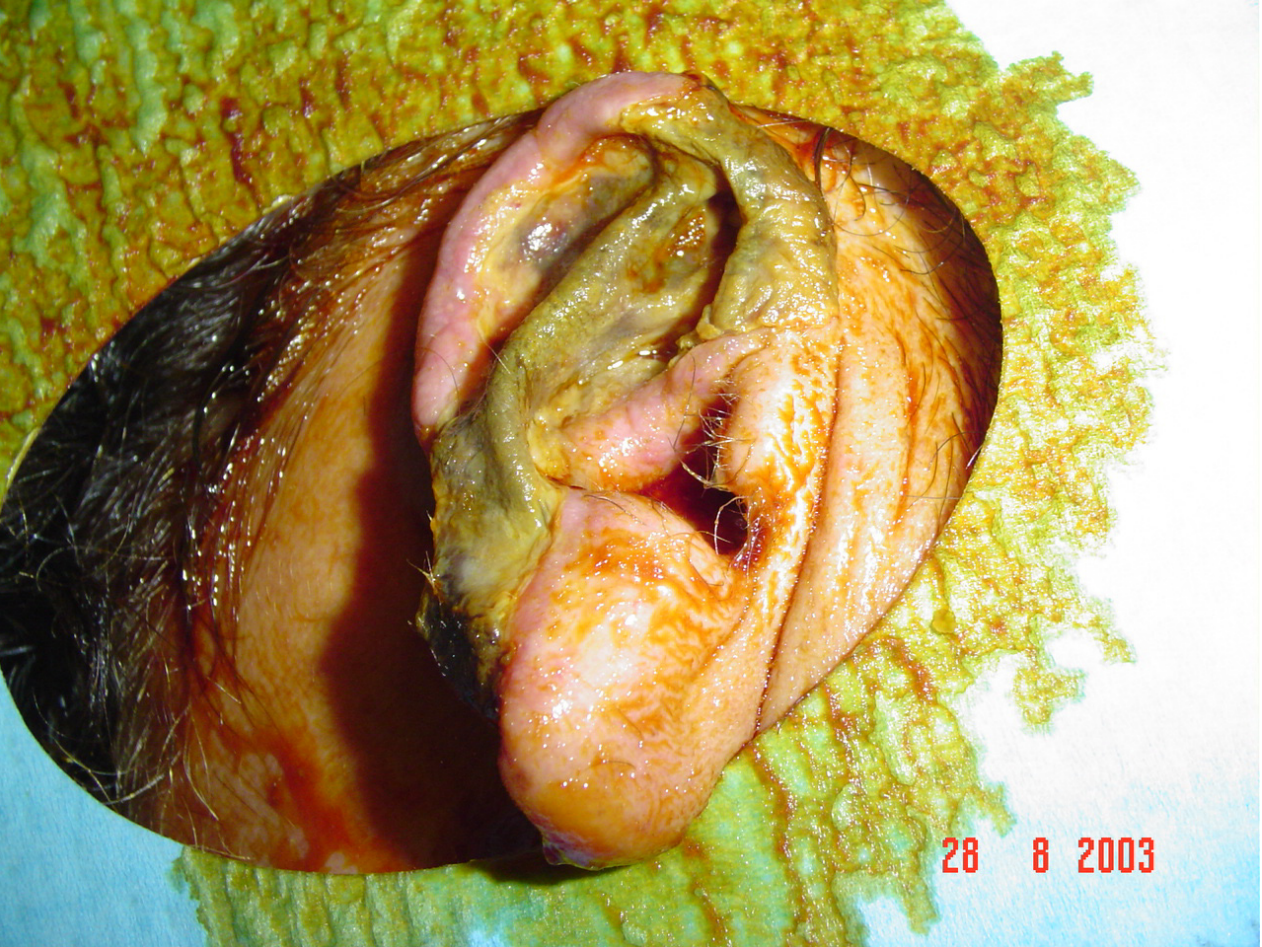
Case 1. Totally replanted right ear on the 21^st ^postoperative day. Satisfactory "take" despite some degree of epidermolysis. Complete reepithelization was noted during the following weeks.

**Figure 3:**
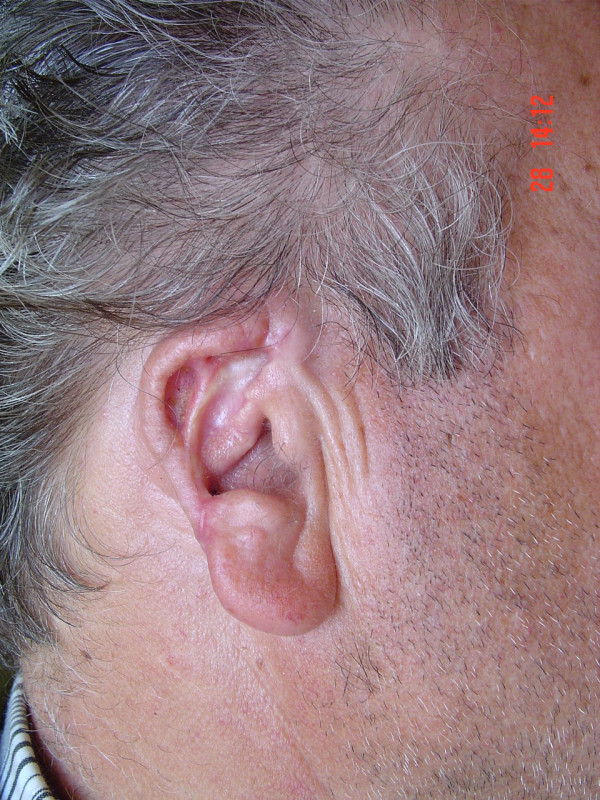
Case 1. Noted an approximately 10% diminishing in the total size of the auricle compared to the normal side, 18 months after surgery.

A 47-year-old male individual was involved in a fight and sustained a human bite resulting in almost a complete amputation of his right ear. Only the ear lobe was left intact. The amputated auricle was placed in a plastic bag with saline, surrounded by ice, and brought to the emergency room with the individual. The patient was immediately started on intravenous antibiotics (Ampicillin/Sulbactam 3 g qid plus metronidazole 500 mg qid), and was led to the operating room approximately four hours following the accident. There, it was decided to reattach the ear as a composite graft. In order to enhance the "take", the epidermis and outer layer of the dermis of the posterior aspect of the graft were sharply excised with a scalpel. In addition, multiple small fenestrations were made in the cartilage and posterior-anterior perichondrium. The skin margin of the amputated stump was dermabraded for a distance of 0.5 mm from the edge and a postauricular flap was elevated. Both the graft and the amputated stump of the ear were meticulously cleaned with rigorous use of normal saline and povidone iodine 10%. No injection of topical vasoconstricting agents was used. The anterior skin of the graft was sutured in layers to the amputated stump of the ear and the skin of the helical rim was sutured to the elevated postauricular flap. Two vicryl 3-0 sutures were used for fixation of the graft to the tissues of the mastoid bed (Figure 1). A Penrose drain was inserted and a loose bandage was applied. The drain was removed three days later and the patient received additional treatment postoperatively with pentoxiphylline orally (400 mg q8h). Antibiotics were administered for a total period of ten days (five days I.V and five days orally). The patient was strongly advised to stop smoking, and was released from hospital on the 7th postoperative day. The ear developed some epidermolysis during the first 3 weeks following surgery but went on to reepithelialize spontaneously (Figure 2). Finally, the replantation was deemed absolutely successful. Three months later, the patient underwent a second operation during which the ear was elevated and the postauricular area was reconstructed with the use of a split-thickness skin graft. No complications have been noted after more than 18 months of follow-up, except of an approximately 10% diminishing in the total size of the auricle compared to the normal side (Figure 3).

### Case 2

**Figure 4:**
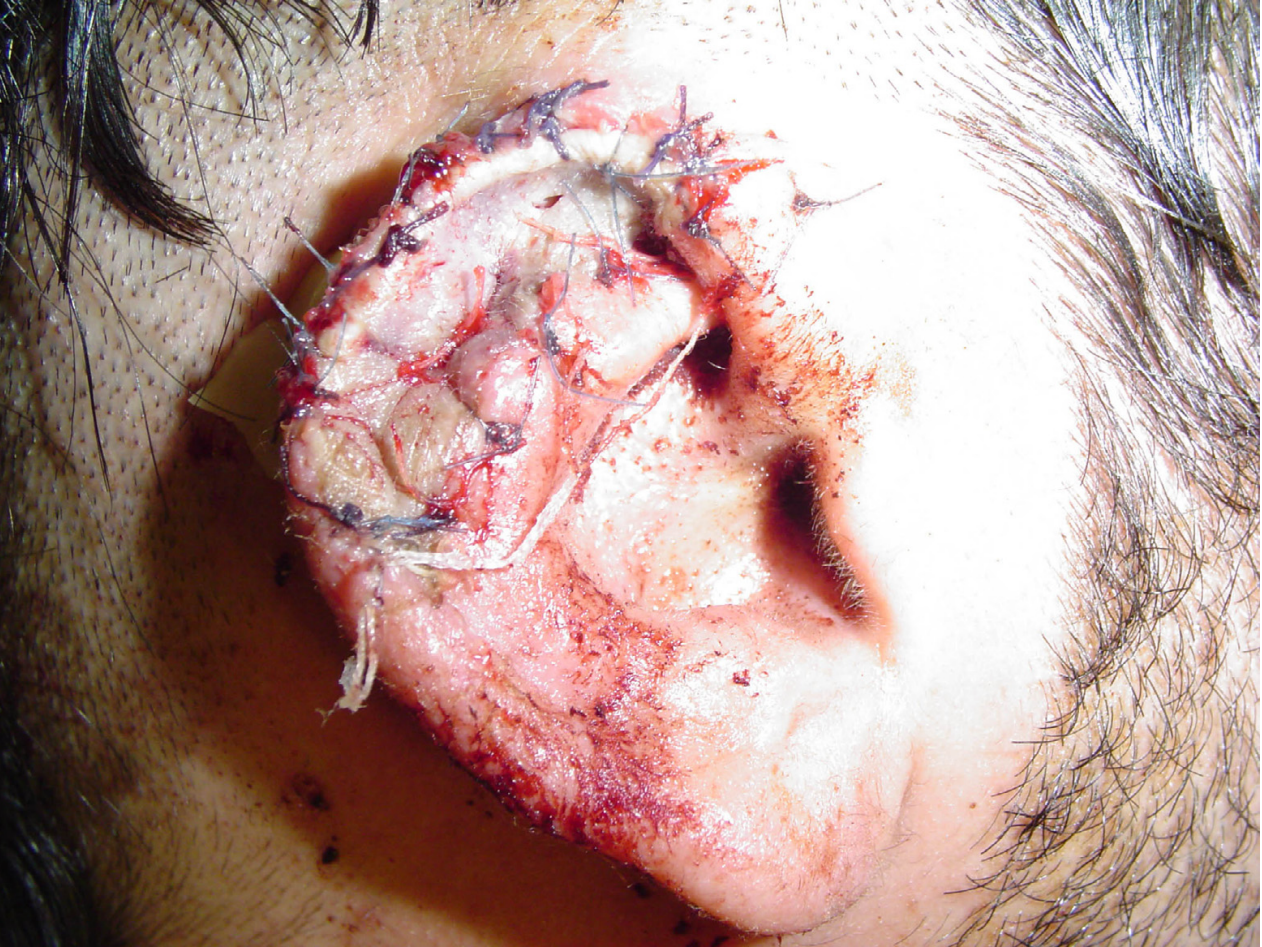
Case 2. Replanted upper one third of the right ear on the 3^rd ^postoperative day. Penrose drain and fixation sutures were removed on that day.

A 20-year-old individual suffered amputation of the superior one third of his right ear after sustaining a human bite during a fight. The amputated part was transferred in the same fashion as for the previous patient and surgery was performed approximately three hours after the injury. The same surgical technique, as described above, was performed and the patient received similar pre- and postoperative therapy. He was released on the 4^th ^postoperative day and three weeks later the survival was deemed very successful (Figure 4). He underwent a second operation for elevation of the ear three months later. No complications have been noted after 4 months of follow-up.

## Results and discussion

Although total or partial traumatic amputation of the ear is a rare occurrence, many treatment modalities have been used up to date [1,4,5]. However, none of them appears to have solved the problem in a definite manner [1,4].

Microsurgical ear replantation was first reported in 1980 and has since proved to be a reliable method for the management of traumatic ear amputation. Successful microsurgical revascularization of amputated auricles has been performed using three different techniques: vein grafts, primary vascular repair, and repair by means of pedicled superficial vessels [2,3]. However, appropriately sized veins are often not available and venous drainage must be accomplished with leech therapy or mechanical drainage and synchronous heparin administration [2,3]. This may result in multiple blood transfusions, with all the associated risks, and prolonged hospitalization [2,3]. Furthermore, microsurgical ear replantation may require lengthy operative time and has a significant failure rate [3]. Finally, the technical complexity of microsurgical operations requires specialized medical personnel, thus not permitting their use in many centers around the world [4,5].

The simple reattachment of the ear as a compound graft usually leads to necrosis and total loss of the organ [1,4]. Therefore, many techniques have been advocated in order to enhance the "take" of a replanted ear [1,4,5]. Some authors have suggested the removal of the skin from the cartilage followed by burial of the cartilage alone under the postauricular skin or at a distance, and reconstruction of the ear in staged fashion [1]. However, the cartilage, denuded of its dermal coverage, becomes distorted due to scarring and the end result after these procedures is not that satisfactory [1].

In 1971, Mladick et al. proposed the principle of the retroauricular pocket, for nonmicrosurgical ear reattachment. This method involved deepithilization of the amputated part, followed by anatomic reattachment to the amputated stump and then burial in a retroauricular pocket [7]. In this way, a larger area of inset and greater surface of contact with the vascular bed was provided for the graft, thus allowing for better composite graft "take" [1,7].

Park et al., described another technique for amputated auricular cartilage burial, by removing all skin from the graft except over the helix area. The denuded cartilage is then sandwiched between a retroauricular flap anteriorly and a facial flap posteriorly. However, the unburied helical skin can undergo necrosis, while three stages are required to achieve a satisfactory result [1,8]. A similar technique has been proposed by Destro and Speranzini, in which all the skin is removed from the graft except over the concha. Multiple small perforations are made in the cartilage which is then covered with a postauricular flap. A second operation is required for elevation of the ear [9].

In cases of more extended trauma with loss of skin of the auricular region, some authors have proposed the use of a platysma myocutaneous flap [4,10]. Mello-Filho et al., have described the implantation of the amputated ear cartilage into the platysma muscle, which is later transferred to its original site in the form of myocutaneous – cartilaginous flap [4]. Finally, other authors have suggested reconstruction of a partial or complete traumatic auricular defects with the use of a free flap from the opposite ear [5,11]. However, these techniques require the use of microsurgery facing the limitations that were earlier mentioned.

We believe that the technique of Baudet et al., whose principles we followed in our cases, is quite simple and very reliable since it allows a great surface of contact between the graft and the vascular bed, substantially increasing its odds of survival. In addition, by maintaining sufficient dermal connection to the cartilage, the latter is protected from distortion due to scarring. In order to enhance revascularization of the graft, we advised our patients to quit smoking and we systematically administered pentoxiphylline. This is an agent that has been shown to improve microcirculation by improving red blood cell elasticity and lowering blood viscocity due to decrease in fibrinogen levels and blood platelet aggregation [12,13].

The graft is always in risk of infection, especially if the mechanism of injury involves a human or animal bite. Therefore antibiotic treatment with good coverage of aerobes and anaerobes of the oral flora is necessary, while the importance of meticulous pro and postoperative care of the amputated auricle and the wound must not be underestimated. On the other hand, long hospital stay can be avoided with the use of the Baudet technique, and, after the first few postoperative days, the individual can be followed on an outpatient basis. However, a second operation will eventually be required for elevation of the ear. The optimal time between the two procedures is unknown. We chose to wait for quite a long time in order to enhance the chance of the graft to survive.

## Conclusion

The Baudet technique has been used successfully in two cases of traumatic ear amputation due to human bites. It is a simple technique, without the need for microsurgery, and produces excellent aesthetic results, while preserving all neighboring tissues in case of failure with subsequent need for another operation.

## Competing interests

The author(s) declare that they have no competing interests.

## Authors' contributions

GV and DK conceived of the study and helped to draft the manuscript. DK performed the surgical operations. AK drafted the manuscript and participated in one operation. JH and CB participated in the surgical operations and patient follow up and helped to draft the manuscript. All authors read and approved the final manuscript.

## References

[B1] Pribaz JJ, Crespo LD, Orgill DP, Pousti TJ, Bartlett RA (1997). Ear replantation without microsurgery. Plast Reconstr Surg.

[B2] Nath RK, Kraemer BA, Azizzadeh A (1998). Complete ear remplantation without venous anastomosis. Microsurgery.

[B3] Kind GM, Buncke GM, Placik OJ, Jansen DA, D' Amore T, Bunche HJ (1997). Total ear replantation. Plast Reconstr Surg.

[B4] Mello-Filho FV, Mamede RCM, Koury AP (1999). Use of a platysma myocutaneous flap for the reimplantation of a severed ear: experience with five cases. Sao Paulo Med J.

[B5] Maral T, Borman H (2000). Reconstruction of the upper portion of the ear by using an ascending helix free flap from the opposite ear. Plast Reconstr Surg.

[B6] Baudet J, Tramond P, Goumain A (1972). A propos d'un procede original de reimplantation pavillon de reille totalement separe [A new technic for the reimplantation of a completely severed auricle]. Ann Chir Plast.

[B7] Mladick RA, Horton CE, Adamson JE, Cohen BI (1971). The pocket principle? A new technique for the reattachment of a severed ear part. Plast Reconstr Surg.

[B8] Park C, Lee CH, Shin KS (1995). An improved burying method for salvaging an amputated auricular cartilage. Plast Reconstr Surg.

[B9] Destro MWB, Speranzini MB (1994). Total reconstruction of the auricle after traumatic amputation. Plast Reconstr Surg.

[B10] Arian S, Chicarelli ZN (1986). Replantation of a totally amputated ear by means of a platysma musculocutaneous "sandwich" flap. Plast Reconstr Surg.

[B11] Sucur D, Ninkovic M, Markovic S, Babovic S (1991). Reconstruction of an avulsed ear by constructing a composite free flap. Br J Plast Surg.

[B12] Adams J, Dhar A, Shukla SD, Silver D (1995). Effect of pentoxifylline on tissue injury and platelet – activating factor production during ischemia – reperfusion injury. J Vas Surg.

[B13] Guerini M, Pecchi S, Rossi C (1983). Effects of pentoxifylline on blood hyperviscocity and peripheral hemodynamics in patients with peripheral obliterating arterial disease. Pharmatherapeutica.

